# Safety of peripheral intravenous administration of hypertonic saline: a systematic review and meta-analysis

**DOI:** 10.3389/fmed.2025.1704530

**Published:** 2025-11-11

**Authors:** Sih-Shiang Huang, Chih-Wei Sung, Chien-Tai Huang, Mu-Yang Hsieh, Jen-Chung Ko, Wang-Huei Sheng, Edward Pei-Chuan Huang

**Affiliations:** 1 Department of Emergency Medicine, National Taiwan University Hospital Hsin-Chu Branch, Hsinchu, Taiwan; 2 Graduate Institute of Clinical Medicine, College of Medicine, National Taiwan University, Taipei, Taiwan; 3 Department of Emergency Medicine, College of Medicine, National Taiwan University, Taipei City, Taiwan; 4 Institute of Epidemiology and Preventive Medicine, College of Public Health, National Taiwan University, Taipei, Taiwan; 5 Division of Cardiology, Department of Internal Medicine, National Taiwan University Hospital Hsin-Chu Branch, Hsinchu, Taiwan; 6 Division of Pulmonology, Department of Internal Medicine, National Taiwan University Hospital Hsin-Chu Branch, Hsinchu, Taiwan; 7 Division of Infectious Diseases, Department of Internal Medicine, National Taiwan University Hospital Hsin-Chu Branch, Hsinchu, Taiwan; 8 School of Medicine, National Taiwan University College of Medicine, Taipei City, Taiwan; 9 Department of Emergency Medicine, National Taiwan University Hospital, Taipei, Taiwan

**Keywords:** hypertonic saline, peripheral administration, infusion-related adverse events, safety, critical care

## Abstract

**Background:**

Hypertonic saline solution is critical for managing symptomatic hyponatremia and increased intracranial pressure (IICP). Though administered via central venous catheters (CVCs) traditionally, peripheral administration is a viable alternative, reducing delays and CVC-associated risks. This review evaluates the safety of peripheral hypertonic saline, focusing on infusion-related adverse events.

**Methods:**

A systematic search of MEDLINE, Embase, and Cochrane Library (up to December 2024) identified studies on peripheral hypertonic saline in adults. Studies reporting phlebitis, infiltration, extravasation, and thrombosis were included. Two reviewers independently screened studies and extracted data. Quality was assessed using the Newcastle-Ottawa Scale and Risk of Bias 2 tool. The review is registered on PROSPERO (CRD42024612330).

**Results:**

Thirteen studies involving 2,354 patients were included, comprising one randomized controlled trial and 12 cohort studies. Quality assessment showed a low risk of bias across all included studies. The pooled incidence of phlebitis was 2.3% (95% CI: 1.2%−4.1%), while infiltration and extravasation occurred at a rate of 2.1% (95% CI: 1.1%−3.9%). Thrombosis was rare, with an incidence of 0.8% (95% CI: 0.3%−1.7%). Most complications were mild and resolved conservatively.

**Conclusion:**

Peripheral hypertonic saline is a safe alternative to CVC placement, particularly in urgent situations where rapid intervention is required. Low complication rates support its broader use in clinical practice, enabling timely treatment while minimizing the risks associated with central access. These findings support consideration for updates to clinical guidelines, advocating for peripheral hypertonic saline as a first-line option in appropriate scenarios to enhance patient outcomes and streamline care delivery.

**Systematic review registration:**

https://www.crd.york.ac.uk/prospero/, identifier: CRD42024612330.

## Introduction

Hypertonic saline, particularly the 3% sodium chloride solution, has emerged as a cornerstone in the treatment of critical conditions such as symptomatic hyponatremia and increased intracranial pressure (IICP) (1, 2). Its clinical utility stems from its ability to correct severe hyponatremia rapidly, thereby mitigating neurological symptoms and preventing severe complications such as seizures and coma (3, 4). Despite its efficacy, the administration of hypertonic saline through a central venous catheter (CVC) has been the traditional practice due to its high osmolarity (approximately 1,026 mOsm/L) and the theoretical risks of local infusion-related adverse events when administered peripherally.

Peripheral intravenous administration of 3% hypertonic saline has garnered attention for its potential to expedite treatment in time-sensitive scenarios where central venous access may not be immediately available (5). Studies have suggested that the complication rates associated with peripheral administration, such as phlebitis, infiltration, and extravasation, range from 3% to 6% and are generally mild (6–8). Furthermore, peripheral intravenous administration mitigates the risks of central line placement, including pneumothorax, thrombosis, and bloodstream infections (9–11). However, concerns persist regarding the safety and tolerability of peripheral veins exposed to hyperosmolar solutions, leading to inconsistent practices and guidelines.

There remains clinical debate regarding on the appropriateness of peripheral 3% hypertonic saline administration. Proponents highlight its practicality in emergency and resource-limited settings, while critics cite the potential for infusion-related injuries and a lack of comprehensive evidence on long-term outcomes. Retrospective and prospective studies have reported low complication rates, but the heterogeneity in study designs, patient populations, and infusion protocols complicates the interpretation and generalization of results.

Given the variability in clinical practice and the paucity of robust, aggregated data, a systematic review and meta-analysis is warranted to consolidate existing evidence. This article aims to provide a definitive assessment of the safety profile of peripheral 3% hypertonic saline administration and its implications for clinical guidelines. By synthesizing data across diverse clinical contexts, this analysis aims to provide a comprehensive evaluation of the safety profile of hypertonic saline when infused through peripheral catheters. The focus is on understanding and quantifying complications such as phlebitis, infiltration, and thrombosis reported in the literature, which may inform future guidelines and improve patient safety in clinical practice.

## Methods

### Study design

This systematic review and meta-analysis were conducted following the Preferred Reporting Items for Systematic Reviews and Meta-Analyses (PRISMA) guidelines and prospectively registered on PROSPERO (CRD42024612330) (12, 13). The aim was to evaluate the safety and adverse events associated with the peripheral administration of hypertonic saline across diverse clinical contexts.

### Search strategy

We performed a comprehensive search of the MEDLINE, Embase, and Cochrane Library databases to identify relevant studies published from their inception until December 2024. No language restrictions were applied during the search. The strategy employed a combination of Medical Subject Headings (MeSH) terms and free-text keywords, including “hypertonic saline,” “sodium chloride,” “peripheral administration,” “adverse events,” and “safety.” Additionally, the references of included studies were manually reviewed to identify further relevant articles. Case reports, case series, and review articles were excluded from the analysis. The complete search strategy is detailed in Supplementary Table 1.

### Eligibility criteria

Studies were included if they reported on the peripheral administration of hypertonic saline of any concentration and provided data on adverse events such as phlebitis, infiltration, extravasation, venous thrombosis, or systemic complications. Eligible studies included randomized controlled trials (RCTs), prospective and retrospective cohort studies that enrolled adult patients (≥18 years). Case reports, case series, review articles, and studies focusing solely on central venous administration were excluded. Non-human studies, or those without safety-related outcomes or sufficient data for analysis were excluded.

### Data extraction and management

Two reviewers (SSH and CWS) independently screened the titles and abstracts, followed by full-text reviews to determine eligibility. Data extraction was conducted using a standardized form that captured key study characteristics (e.g., design, population, and setting), details of the intervention (e.g., concentration of hypertonic saline, indication for hypertonic saline, infusion rate, duration of therapy, gauge of the peripheral catheter), and outcomes (e.g., incidence of adverse events). Any discrepancies between the reviewers were resolved through discussion or, when necessary, consultation with a third reviewer (CWS).

### Quality assessment

The quality of included studies was assessed using the Newcastle-Ottawa Scale (NOS) for non-randomized studies and the Risk of Bias 2 (ROB2) tool for RCTs (14, 15). NOS evaluated selection, comparability, and outcome domains, while ROB2 focused on bias arising from randomization, deviations from intended interventions, missing outcome data, measurement of outcomes, and selection of reported results. Studies were rated as low, moderate, or high risk of bias.

### Statistical analysis

A meta-analysis was conducted using a random-effects model to account for heterogeneity across studies. The primary outcome was the pooled incidence of adverse events, including phlebitis, infiltration, extravasation and thrombosis, expressed as proportions with 95% confidence intervals (CIs). Heterogeneity was assessed using the *I*^2^ statistic, with thresholds of 25%, 50%, and 75% indicating low, moderate, and high heterogeneity, respectively. Funnel plots was used to assess publication bias.

## Result

### Selection process and quality assessment of included studies

**Figure 1:**
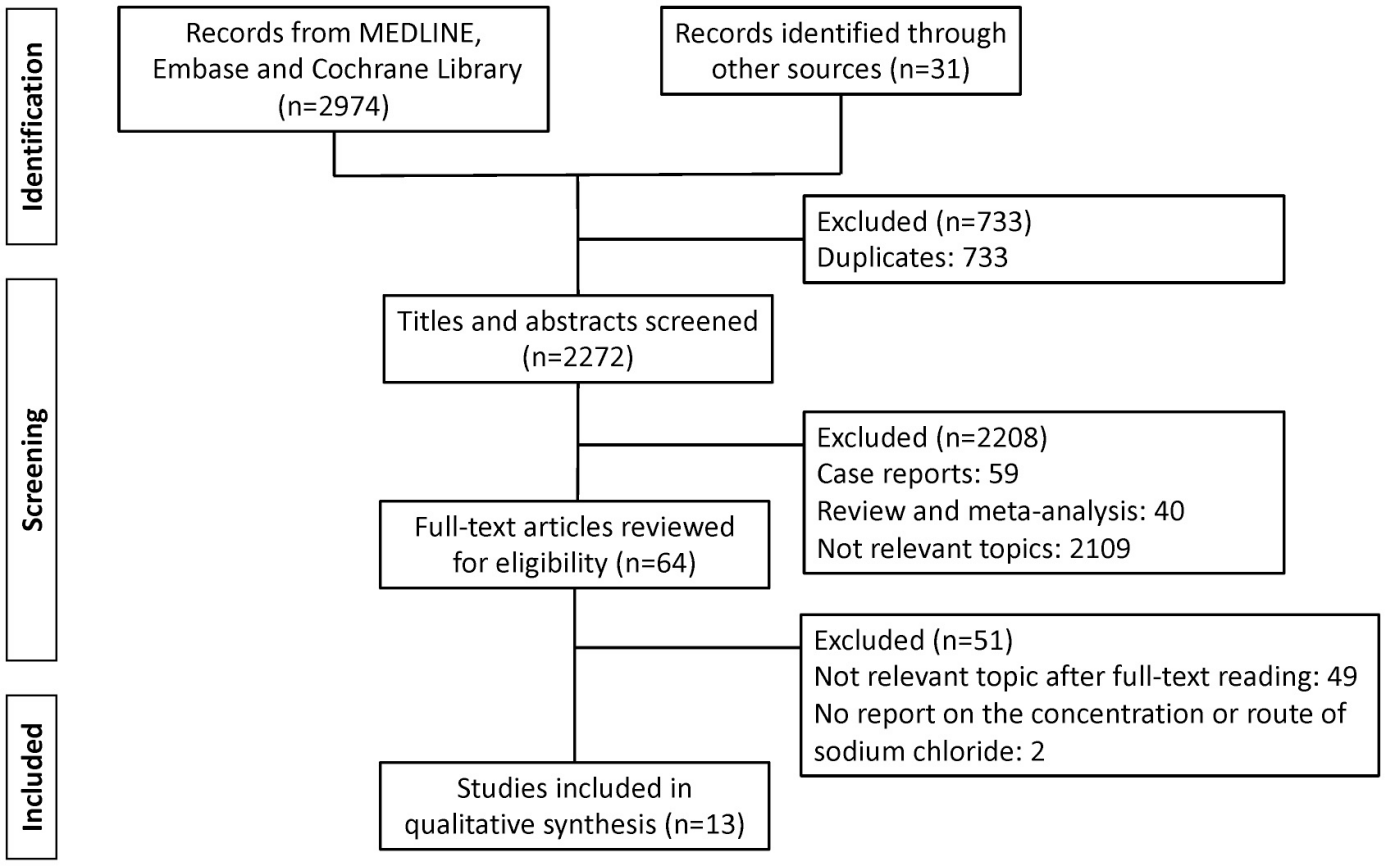
Flow diagram depicting the study selection process for the systematic review and meta-analysis, following the Preferred Reporting Items for Systematic Reviews and Meta-Analyses (PRISMA) guidelines.

Figure 1 illustrates the flow of records through the study selection process. A total of 3,005 records were identified through the initial search, comprising 2,974 records from Medline and Embase and 31 records identified through other sources, including manual search of the reference. After removing 733 duplicates, 2,272 records remained for title and abstract screening. During the screening process, 2,208 records were excluded due to irrelevance to the study topic (*n* = 2,109), case reports (*n* = 59), or review articles/meta-analyses (*n* = 40). Subsequently, 64 full-text articles were assessed for eligibility, of which 51 were excluded: 49 were deemed irrelevant to the study topic upon full-text review, and 2 did not report either the concentration or the route of sodium chloride administration. Ultimately, 13 studies met the inclusion criteria and were included in the qualitative synthesis (6, 7, 16–26).

Supplementary Figures 1, 2 illustrate the quality assessment of the included studies. Supplementary Figure 1 evaluates 12 non-randomized studies across three domains: selection, comparability, and outcome, with no high risk of bias noted. Supplementary Figure 2 assesses the sole RCT included in the meta-analysis, covering five risk of bias domains. The RCT demonstrated a low risk of bias in all domains except for some concerns noted in deviations from the intended intervention. Overall, no high risk of bias was identified across all included studies.

### Characteristics of included studies

Table 1 provides an overview of the 13 included studies, highlighting their design, patient population, infusion attempts, sodium chloride concentrations, indication for hypertonic saline, infusion rates, duration of therapy, catheter gauge, and reported adverse events. The majority of the studies were retrospective in nature, with prospective studies and one randomized controlled trial also represented. Sodium chloride concentrations ranged from 2% to 23.4%, with the most common concentration being 3%. The clinical indications for hypertonic saline varied across studies, most commonly including intracranial hypertension and symptomatic hyponatremia. Reported infusion rates ranged from slow continuous infusions of 30–50 mL/h to rapid bolus administrations up to 999 mL/h, reflecting differences in institutional protocols and treatment purposes. Catheter gauges varied across studies, reflecting differences in clinical practices and patient-specific requirements. Adverse events such as phlebitis, infiltration, and thrombosis were commonly reported, with variability in incidence potentially linked to catheter size, infusion protocol, and study design.

**Table 1:** Summary of studies evaluating the peripheral administration of hypertonic saline and associated outcomes.

**Authors**	**Country**	**Study design**	**Number of patients included**	**Number of infusion attempts**	**Concentration of sodium chloride**	**Indication for hypertonic saline**	**Infusion rate**	**Duration of therapy**	**Peripheral intravenous catheter gauge**	**Reported adverse events**
Perez and Figueroa (6)	United States	Prospective	28	34	3%	Intracranial hypertension, hyponatremia	Range 30–50 mL/h	Mean 47 h	From 16 to 20	Phlebitis, infiltration, thrombosis
Jones et al. (16)	United States	Retrospective	213	213	3%	Not specified	Maximum 75 mL/h	Median 44 h	16 (3.8%), 18 (47.4%), 20 (46.5%), 22 (2.3%)	Phlebitis, extravasation, thrombosis
Meng et al. (17)	United States	Prospective	60	106	3%	Not specified	Maximum 100 mL/h	< 100 h	Median 20 [IQR 18–20]	Phlebitis
Dillon et al. (7)	United States	Retrospective	66	168	3%	Hyponatremia, cerebral edema	Maximum 50 mL/h	Mean 58 h	20 or lower (60%) 22 or higher (40%)	Phlebitis, infiltration
Mesghali et al. (18)	United States	Retrospective	85	85	3%	Traumatic brain injury	Not reported	Not reported	Not reported	Extravasation
Baek et al. (19)	South Korea	Randomized controlled trial	175	175	3%	Hyponatremia	Rapid intermittent bolus and slow continuous infusion using a weight-based protocol	< 48 h	Not reported	Phlebitis
Jannotta et al. (20)	United States	Prospective	103	103	3%	Intracranial hypertension, hyponatremia	Maximum 100 mL/h	< 72 h	From 14 to 22, with majority being 18–20 (85%)	Phlebitis
Faiver et al. (21)	United States	Retrospective	51	57	23.4%	Ongoing cerebral herniation, Intracranial hypertension	Bolus 30 mL ≥10 min	Bolus	Not reported	Extravasation
Brown et al. (22)	United States	Retrospective	37	37	3%	Neurologic emergencies	Bolus 250 mL (760 mL/h)	Bolus	Median 18 [IQR 18–20]	Phlebitis, extravasation
Deveau et al. (23)	United States	Retrospective	422	706	3%	Intracranial hypertension, hyponatremia	Median 30 mL/h	Not reported	14 (0.1%), 16 (2.1%), 18 (20%), 20 (49%), 22 (10.9%), 24 (0.1%), Undocumented (17.7%)	Phlebitis, infiltration
Shafi and Muath (24)	Saudi Arabia	Prospective	43	97	2% (51%) 3% (49%)	Neuro emergencies	Median 50 mL/h	Mean 52 h	18 (55.8%) 20 (44.2%)	Phlebitis, extravasation
Dumont et al. (25)	United States	Retrospective	199	199	2% (42.7%) 3% (57.3%)	Intracranial hypertension, hyponatremia	Maximum 50 mL/h	Median 37 h	16 (1.5%) 18 (10.6%) 20 (69.8%) 22 (14.6%) 24 (2.5%)	Phlebitis
Khasiyev et al. (26)	United States	Retrospective	124	216	3%	Intracranial hypertension	Bolus 250 mL (999 mL/h)	Bolus	18 or 20	Phlebitis, infiltration, thrombosis

Some rarely reported outcomes, such as pain at the injection site, electrolyte imbalances, and hypotension, were excluded from our meta-analysis due to the limited number of studies reporting these outcomes, which did not meet the minimum threshold typically required for meta-analysis (commonly three or more studies).

### Analysis of safety outcomes

The safety outcomes of phlebitis, infiltration, and thrombosis were analyzed using a random-effects model to account for variability among studies. Phlebitis was reported in 12 studies, infiltration or extravasation in 11 studies, and thrombosis in 5 studies.

**Figure 2:**
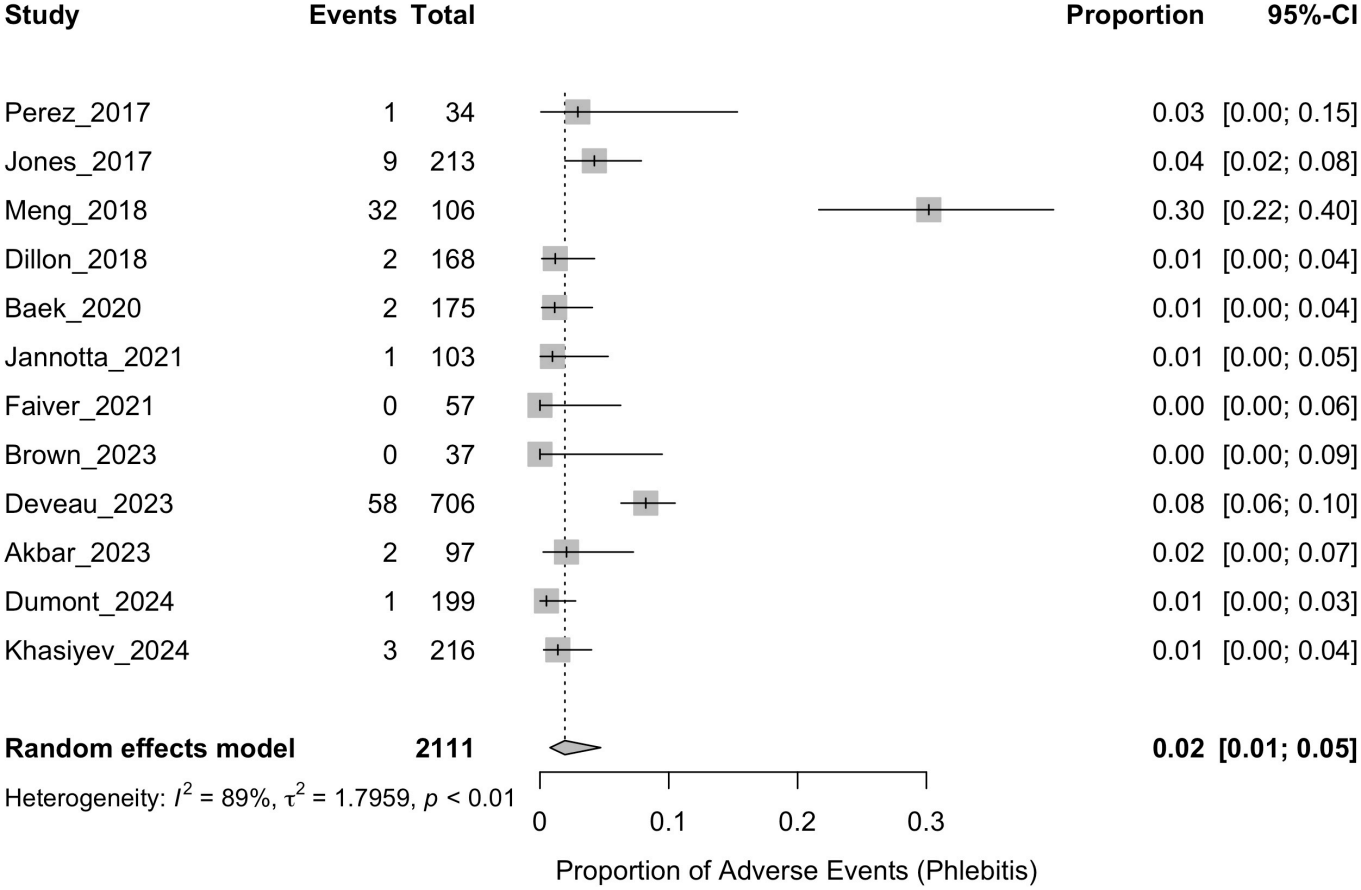
Forest plot summarizing the proportion of phlebitis events reported in studies examining the safety of peripheral administration of hypertonic saline. Each row corresponds to an individual study, with events and total participants listed. The squares represent the proportion of events, with the size of the square indicating the weight of the study in the meta-analysis. Horizontal lines depict the 95% confidence intervals (CI) for each study. The diamond at the bottom of the plot represents the overall pooled proportion, using a random-effects model.

**Figure 3:**
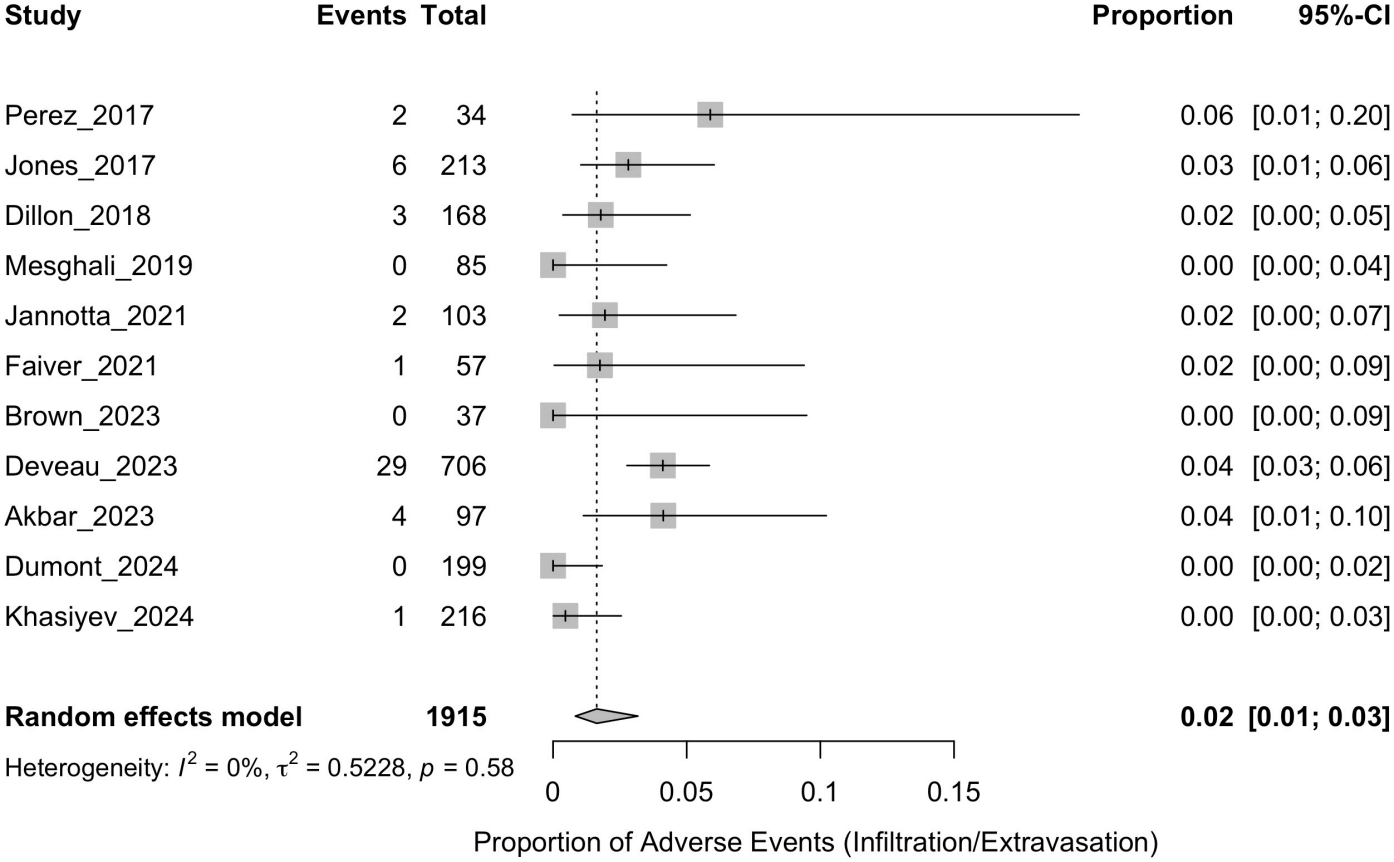
Forest plot summarizing the proportion of infiltration/extravasation events reported in studies examining the safety of peripheral administration of hypertonic saline. Each row corresponds to an individual study, with events and total participants listed. The squares represent the proportion of events, with the size of the square indicating the weight of the study in the meta-analysis. Horizontal lines depict the 95% confidence intervals (CI) for each study. The diamond at the bottom of the plot represents the overall pooled proportion, using a random-effects model.

**Figure 4:**
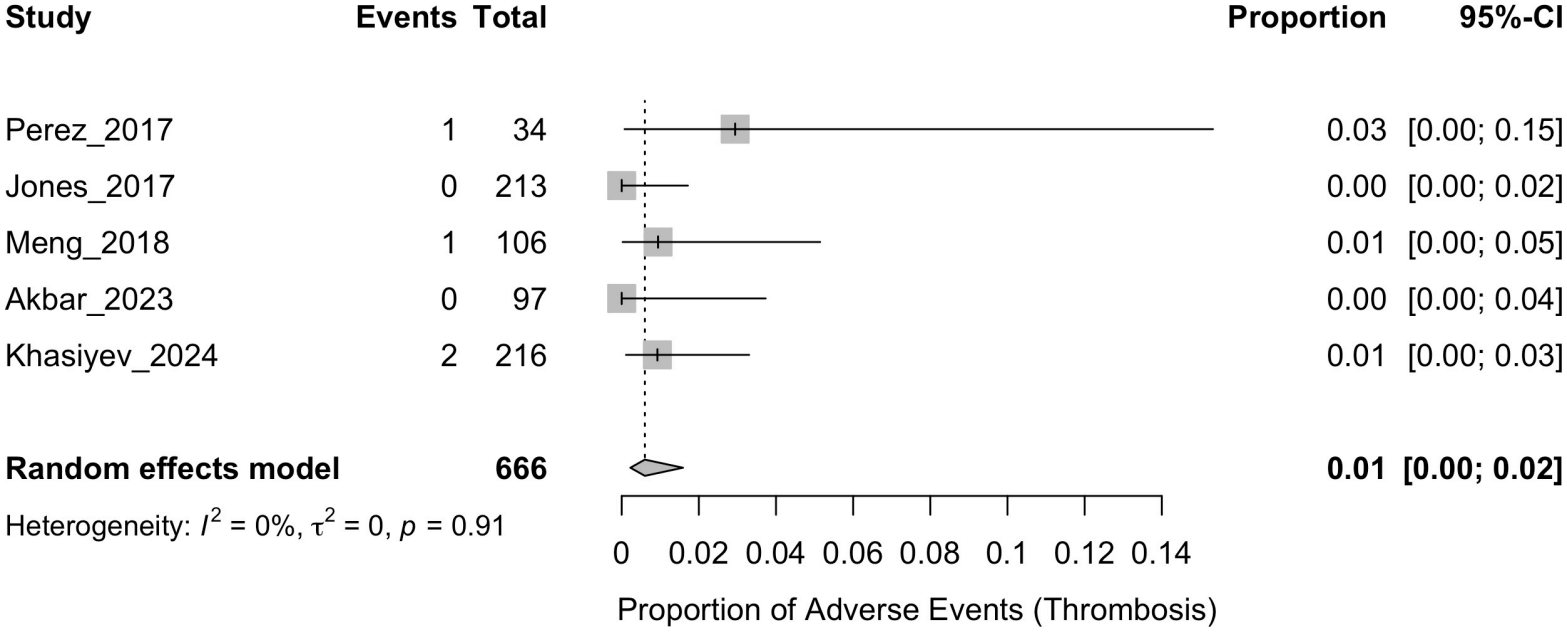
Forest plot summarizing the proportion of thrombosis events reported in studies examining the safety of peripheral administration of hypertonic saline. Each row corresponds to an individual study, with events and total participants listed. The squares represent the proportion of events, with the size of the square indicating the weight of the study in the meta-analysis. Horizontal lines depict the 95% confidence intervals (CI) for each study. The diamond at the bottom of the plot represents the overall pooled proportion, using a random-effects model.

The pooled incidence of phlebitis is presented in Figure 2. The analysis demonstrated a pooled event rate of 0.02 (95% CI: 0.01–0.05), with significant heterogeneity (*I*^2^ = 89%, *p* < 0.01), indicating considerable variability among the included studies. For infiltration/extravasation, the results are displayed in Figure 3, showing a pooled incidence of 0.02 (95% CI: 0.01–0.03). The heterogeneity across the studies was low (*I*^2^ = 0%, *p* = 0.58), suggesting consistent findings across studies. Thrombosis analysis, shown in Figure 4, reported a pooled incidence of 0.01 (95% CI: 0.00–0.02). The heterogeneity was also low (*I*^2^ = 0%, *p* = 0.91), indicating stability in the outcomes.

### Evaluation of publication bias

Supplementary Figure 3 presents the funnel plot of the included studies to evaluate publication bias. The plot demonstrates a symmetrical distribution of studies around the pooled estimate, suggesting minimal evidence of publication bias. Smaller studies with larger standard errors are more scattered toward the base, while larger studies converge toward the top, forming a typical funnel shape. This pattern indicates that the meta-analysis findings are unlikely to be skewed by selective reporting or missing studies.

## Discussion

This systematic review and meta-analysis demonstrate that peripheral administration of 3% hypertonic saline is a safe and effective option for managing critical conditions such as hyponatremia and IICP. Our findings align with the growing body of evidence supporting the peripheral route as a viable alternative to CVC administration, especially in emergency scenarios where time is crucial.

### Expanded safety evidence

Unlike previous analyses, such as Madieh et al., which included case series and other less rigorous study designs, our review exclusively integrated original studies with detailed safety outcomes (8). This methodological refinement enabled a more robust evaluation of safety profiles. Importantly, our analysis included a larger population base compared to prior studies, specifically doubling the sample size for complications like phlebitis and infiltration and increasing it 6 fold for thrombosis-related complications. This expanded dataset revealed lower incidences of phlebitis and infiltration than those reported in Madieh et al. Specifically, our study reported a phlebitis incidence of 0.02 compared to 0.06 in Madieh's study. For infiltration, our incidence was 0.02, while Madieh et al. reported 0.033. Both studies reported a thrombosis incidence of 0.01, reaffirming the safety of peripheral hypertonic saline administration. Taken together, the pooled results indicate that adverse event rates associated with peripheral hypertonic saline remain within clinically acceptable limits. The consistency of findings and the absence of publication bias enhance the credibility of the synthesized evidence.

### Grading of phlebitis

Most of the studies included in this analysis utilized the Visual Infusion Phlebitis (VIP) Scale as a reference for assessing phlebitis (27). Notably, the majority of reported phlebitis cases were graded as score 1 or 2 on this scale, indicating mild symptoms such as pain at the catheter site or minimal swelling and erythema. This underscores that the observed phlebitis cases were predominantly mild and clinically manageable, further supporting the safety of peripheral administration of hypertonic saline.

### Peripheral administration vs. CVC

Comparing peripheral administration with the CVC route, our findings underscore the significantly higher complication rates associated with CVCs. Moreover, the lower complication rates observed with peripheral administration of hypertonic saline are noteworthy compared to the CVC route. While complications such as phlebitis or infiltration may occur, they are generally mild and manageable through simple interventions such as catheter repositioning. This contrasts starkly with CVC-related complications, which are not only more frequent but also more severe and difficult to manage. For example, arterial puncture or pneumothorax, as highlighted by McGee et al., present significant clinical challenges and require specialized intervention, increasing both patient risk and healthcare burden (28). Peripheral administration, therefore, provides a safer alternative with fewer safety concerns and comparable efficacy in critical scenarios.

### Variability across studies

Our analysis also considered variability in complication rates among studies. For instance, Meng et al. reported higher incidences of phlebitis and infiltration compared to other studies (17). However, these complications were not significantly more frequent than those observed with standard intravenous solutions in the control arms. Notably, Meng et al. employed the VIP Scale from the Infusion Therapy Standards of Practice, ensuring standardized criteria across studies. This consistency supports the conclusion that peripheral administration of hypertonic saline poses no greater risk than standard fluid administration. Further evidence from Faiver et al. strengthens this assertion (21). Despite using a more concentrated 23.4% hypertonic saline solution, their study documented no phlebitis and only one case of infiltration among 57 patients. Similarly, Alenazi et al. reviewed seven studies, including one case series and one case report, reporting low incidences of complications with peripheral administration of 3% saline (29). These findings collectively reinforce the safety of peripheral administration even with higher solution concentrations.

### Practical management of complications

As discussed earlier, CVC carries substantially higher risks, including mechanical and infectious complications, whereas the complications associated with peripheral hypertonic saline administration are typically minor and self-limited. For complications such as infiltration or phlebitis, the management strategy typically involves repositioning the catheter to a different peripheral site. This straightforward approach effectively resolves most adverse events, maintaining the low overall complication rate. Thus, while minor complications may arise, they are manageable and do not outweigh the benefits of peripheral administration in acute care settings.

### Emergency use and efficiency

In emergencies such as symptomatic hyponatremia or IICP, the ability to rapidly and efficiently administer hypertonic saline through peripheral lines is invaluable. Recent evidence underscores the risks associated with excessively slow correction of hyponatremia, which has been linked to increased mortality and prolonged hospital stays. A systematic review and meta-analysis revealed that slow and very slow correction rates (< 8 mEq/L or < 4–6 mEq/L per 24 h, respectively) were associated with significantly higher in-hospital and 30-day mortality compared to more rapid correction (≥8–10 mEq/L per 24 h) (30).

For managing IICP, timely intervention is critical. Hypertonic saline has demonstrated comparable efficacy to mannitol in reducing IICP while posing a lower risk of hypotension—a significant concern in emergency settings where patients are often hemodynamically unstable. Notably, hypertonic saline achieves effective ICP reduction and avoids the intravascular volume depletion associated with mannitol. This distinction further supports its preferential use in critical care scenarios. The ability to administer hypertonic saline safely through peripheral lines also ensures that emergency interventions can be implemented promptly, particularly when central access is delayed or impractical (31).

While our findings support the overall safety of peripheral administration in critically ill patients, it is important to consider variation in needs across different subgroups. For example, patients in neurocritical care—such as those with traumatic brain injury or cerebral edema—may require more frequent or prolonged hypertonic therapy, which could increase the cumulative risk of infusion-related complications through peripheral access. In contrast, general critical care patients, including those with acute symptomatic hyponatremia, often benefit from shorter courses of hypertonic saline, making peripheral administration both safer and more feasible in this context. Despite these theoretical concerns, recent studies have shown that even prolonged peripheral infusion of 3% hypertonic saline at relatively high rates appears to be safe when appropriately monitored (29, 32). Tailoring the route of administration to the anticipated duration and intensity of therapy is essential to optimize outcomes across diverse critical care populations.

These findings emphasize the necessity of a balanced and evidence-based approach. The efficiency and safety of peripheral hypertonic saline administration enable timely correction, reducing the risks of delayed treatment. This strategy not only improves patient outcomes but also mitigates the strain on healthcare resources. Ultimately, the judicious use of hypertonic saline in acute care settings, especially when central access is unavailable, exemplifies proactive and effective management.

### Limitations

However, some limitations must be acknowledged. High heterogeneity was observed in the analysis of phlebitis, warranting cautious interpretation of these results. Additionally, only one randomized controlled trial was included, emphasizing the need for further high-quality RCTs to strengthen the evidence base. Variability in catheter sizes and infusion rates, with some studies lacking detailed reporting, also precluded subgroup analyses. Future research should address these factors with standardized protocols to provide more conclusive evidence.

## Conclusions

Peripheral administration of 3% hypertonic saline is a safe and effective option for managing symptomatic hyponatremia and IICP. The low incidence of mild, manageable complications and its practicality in emergencies underscore its value, especially when central access is delayed. These findings support its integration into clinical practice, while further well-designed studies are warranted to refine guidelines and optimize patient outcomes in diverse clinical settings.

## Data Availability

The original contributions presented in the study are included in the article/Supplementary material, further inquiries can be directed to the corresponding author.

## Supplementary material

Supplementary Figure 1 Bar plot illustrating the Newcastle-Ottawa Scale (NOS) scores for each study included in the systematic review and meta-analysis. The NOS assesses the quality of non-randomized studies based on three domains: selection (red), comparability (green), and outcome (blue). Each bar represents the total NOS score for an individual study, with segments color-coded to show contributions from the three domains. Studies are ordered by year of publication.

Supplementary Figure 2 Risk of Bias (RoB2) evaluation, assessed across five domains: D1 (bias arising from the randomization process), D2 (bias due to deviations from intended interventions), D3 (bias due to missing outcome data), D4 (bias in measurement of the outcome), and D5 (bias in selection of the reported result). The green circles represent a “Low” risk of bias, while the yellow circle represents "Some concerns.

Supplementary Figure 3 Funnel plot assessing potential publication bias in the meta-analysis of studies reporting adverse events following peripheral administration of hypertonic saline. The x-axis represents the logit-transformed proportion of adverse events, while the y-axis shows the standard error. Each point represents an individual study included in the meta-analysis. The dotted vertical line indicates the pooled estimate of the logit-transformed proportion, and the diagonal dashed lines represent the pseudo 95% confidence limits.

Supplementary Table 1 Search strategy and criteria for systematic review and meta-analysis on peripheral administration of hypertonic saline.
