# Supplementary figures and images for: Safety of peripheral intravenous administration of hypertonic saline: a systematic review and meta-analysis
Source: Front Med (Lausanne). 2025 Nov 11;12:1704530. doi: 10.3389/fmed.2025.1704530 (PMC12644001; doi:10.3389/fmed.2025.1704530)

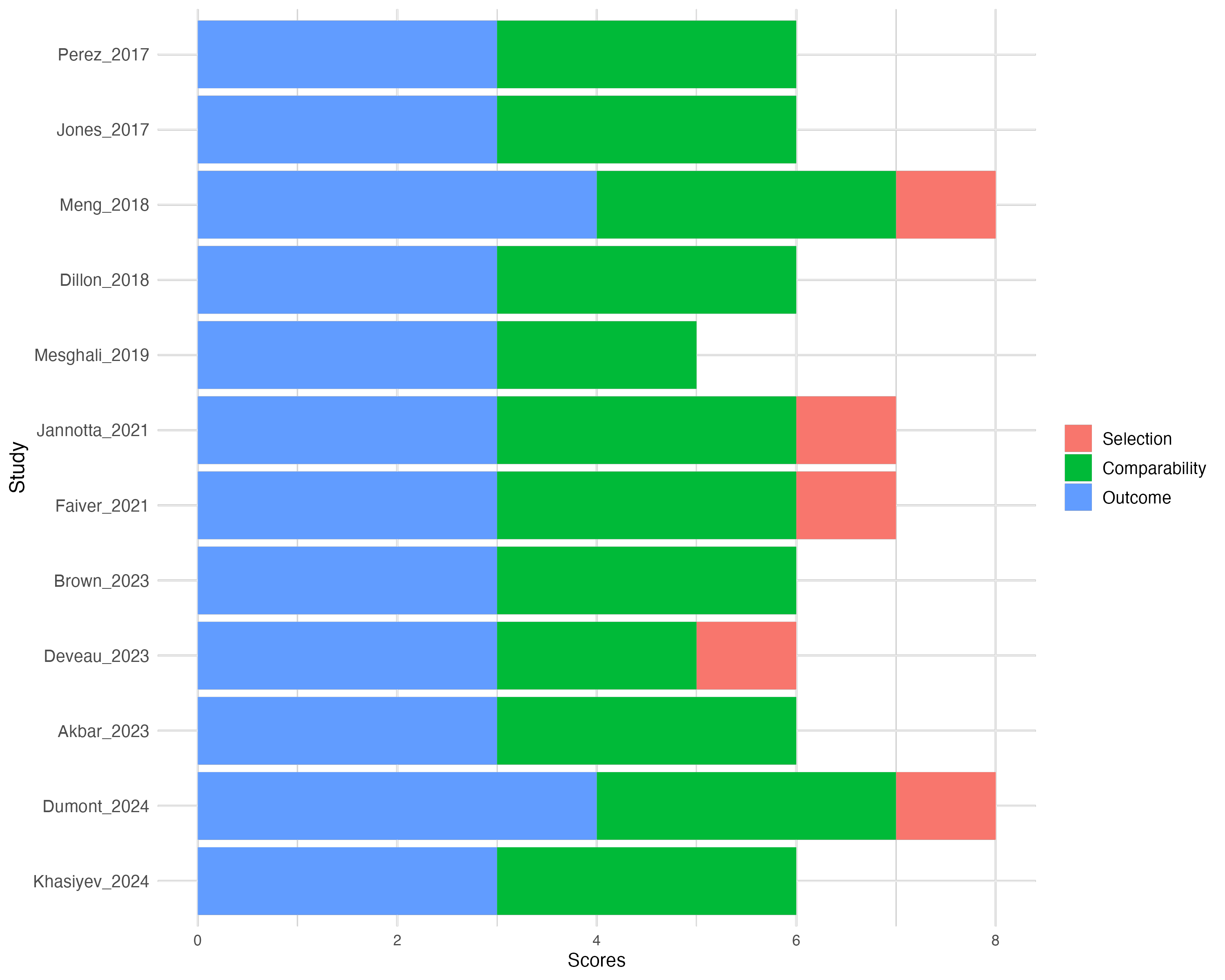

Supplement: Supplementary Figure 1 — Bar plot illustrating the Newcastle-Ottawa Scale (NOS) scores for each study included in the systematic review and meta-analysis. The NOS assesses the quality of non-randomized studies based on three domains: selection (red), comparability (green), and outcome (blue). Each bar represents the total NOS score for an individual study, with segments color-coded to show contributions from the three domains. Studies are ordered by year of publication. [file Image_1.tiff]

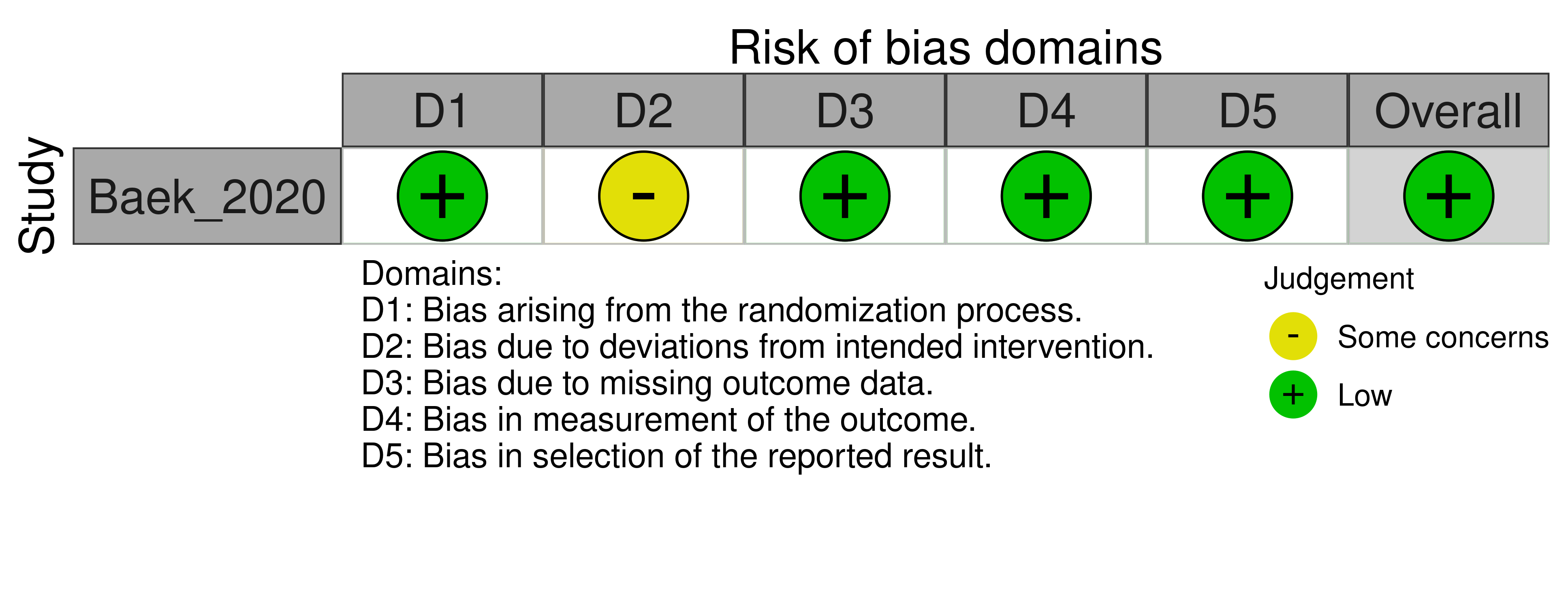

Supplement: Supplementary Figure 2 — Risk of Bias (RoB2) evaluation, assessed across five domains: D1 (bias arising from the randomization process), D2 (bias due to deviations from intended interventions), D3 (bias due to missing outcome data), D4 (bias in measurement of the outcome), and D5 (bias in selection of the reported result). The green circles represent a “Low” risk of bias, while the yellow circle represents "Some concerns. [file Image_2.tiff]

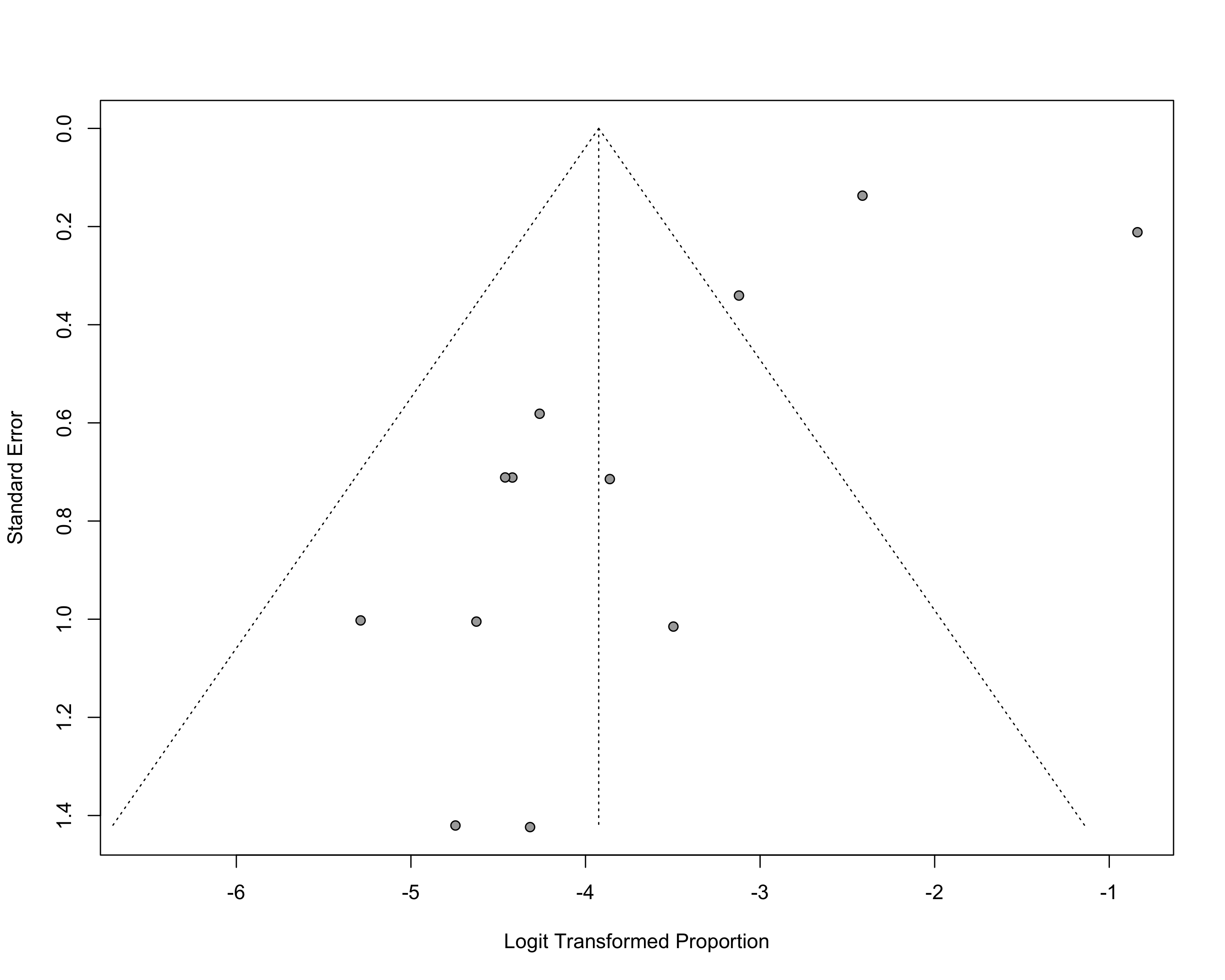

Supplement: Supplementary Figure 3 — Funnel plot assessing potential publication bias in the meta-analysis of studies reporting adverse events following peripheral administration of hypertonic saline. The x-axis represents the logit-transformed proportion of adverse events, while the y-axis shows the standard error. Each point represents an individual study included in the meta-analysis. The dotted vertical line indicates the pooled estimate of the logit-transformed proportion, and the diagonal dashed lines represent the pseudo 95% confidence limits. [file Image_3.tiff]
