# Supplementary material for: Safety of peripheral intravenous administration of hypertonic saline: a systematic review and meta-analysis
Source: Front Med (Lausanne). 2025 Nov 11;12:1704530. doi: 10.3389/fmed.2025.1704530 (PMC12644001; doi:10.3389/fmed.2025.1704530)
Supplement: Supplementary Table 1 — Search strategy and criteria for systematic review and meta-analysis on peripheral administration of hypertonic saline. [file Table_1.docx]

| Supplementary table 1. Search strategy and criteria for systematic review and meta-analysis on peripheral administration of hypertonic saline | |
| --- | --- |
| Criterion | Detail |
| Search terms | ("Sodium Chloride, Hypertonic"[Mesh] OR "hypertonic saline" OR "3% saline" OR "high-concentration saline")  AND  ("Peripheral Catheterization"[Mesh] OR "peripheral venous catheter" OR "peripheral line" OR "peripheral vein" OR "intravenous peripheral" OR "PIV" OR "IV line")  AND  ("Treatment Outcome"[Mesh] OR "Adverse Effects"[Subheading] OR "safety" OR "adverse events" OR "phlebitis" OR "extravasation" OR "complications" OR "adverse effects" OR "toxicity") |
| Language | No restrictions |
| Timeframe | From inception to January 2025 |
| Databases | Medline, Embase, Cochrane Library |
| Inclusion criteria | Articles investigating the safety outcome of peripheral administrated hypertonic saline |
